# International Urogynecological Consultation Chapter 2.2: Imaging in the Diagnosis of Pelvic Organ Prolapse

**DOI:** 10.1007/s00192-024-05948-4

**Published:** 2025-03-26

**Authors:** Lioudmila Lipetskaia, Ankita Gupta, Rachel Y. K. Cheung, Vik Khullar, Sharif Ismail, Megan Bradley, Roopali Karmakar, Shari Clifton, Josephine Doo, Lieschen Quiroz

**Affiliations:** 1 Division of Urogynecology, Department of Obstetrics and Gynecology, Cooper Medical School of Rowan University, 402 E Oak Ave, Moorestown, USA; 2 Division of Urogynecology, Department of Obstetrics and Gynecology, University of Louisville, Louisville, USA; 3 Department of Obstetrics and Gynaecology, The Chinese University of Hong Kong | CUHK, Ma Liu Shui, Hong Kong; 4 Department of Urogynaecology, Imperial College London, London, UK; 5 University Hospitals Sussex National Health Service (NHS) Foundation Trust/Brighton and Sussex Medical School, Brighton, UK; 6 Division of Urogynecology, Department of Obstetrics and Gynecology, University of Pittsburgh, Pittsburgh, PA USA; 7 Department of Obstetrics and Gynaecology, Chelsea and Westminster NHS Foundation Trust, West Middlesex University Hospital, Isleworth, UK; 8 Division of FPMRS, Department of Obstetrics and Gynecology, University of Oklahoma Health Sciences Center, Oklahoma City, OK USA

**Keywords:** Imaging, Ultrasound, MRI, CT scan, Fluoroscopy, Pelvic organ prolapse, Diagnosis

## Abstract

**Introduction and Hypothesis:**

This section of Chapter 2.2 of the International Urogynecology Consultation on Pelvic Organ Prolapse (POP), reviews the literature on the role of imaging in the diagnosis of POP.

**Methods:**

An international group of nine urogynecologists and one university-based medical librarian adhered to the framework of the scoping review. The group performed a search of the literature using pre-specified search terms in Scopus, OVID Medline, and PubMed. Publications were eliminated if not relevant to the diagnostic value of POP imaging. The remaining articles were evaluated for quality using the Joanna Briggs Institute Checklist for Diagnostic Test Accuracy Studies. The resulting list of articles was used to perform a comprehensive narrative review of the diagnostic value of imaging modalities for the diagnosis of POP.

**Results:**

The original search yielded 3,289 references, 135 of which were used by the writing group.

**Conclusions:**

Most imaging studies utilized in the diagnoses of POP lacked standardization in the definition of POP. Most imaging studies lack standardization in the protocols used to diagnose POP within each imaging technique. Ultrasound- and MRI-related studies are most represented in the literature, compared with fewer CT- and X-ray-/fluoroscopy-related studies. Therefore, radiographic imaging is of limited value in the diagnosis of POP.

## Introduction

This report is part of the series of articles that are produced by the International Urogynecological Consultation (IUC), a project sponsored by the International Urogynecology Association (IUGA) on the management of pelvic organ prolapse (POP). This is a four-chapter project with 16 reports. The present article is from the second chapter reporting on the evaluation of POP. It focuses on the role of imaging in the diagnosis of POP. POP is defined as the descent of any one or more of the vaginal walls, cervix, or vaginal vault after hysterectomy [1]. The correlation of this examination finding with the symptom of being able to see or feel a vaginal bulge is necessary for the diagnosis of POP. This relationship mostly happens at or below the level of the hymenal plane. Chapter 1.1 of the IUC evaluated the definition of POP and stressed that it should only be made in a patient with the complaint of a vaginal bulge or in a patient with a medically morbid condition directly related to POP [2]. As symptoms play a major role in the diagnosis it can be difficult to appreciate various symptoms and the diagnosis becomes more complex when the patient’s symptoms are disproportionate to the level of descent seen on examination. It has been postulated that imaging techniques, such as ultrasound or magnetic resonance imaging (MRI), can provide additional information to assist in those instances where the diagnosis is not straightforward [3, 4]. Imaging techniques can show and measure the degree of the displacement of pelvic organs and their descent against a defined reference point. Hence, imaging can assist in both the diagnosis and quantification of prolapse. For example, the reference points commonly used to assess POP on MRI are the pubo-coccygeal line (PCL) and midpubic line (MPL), which are fixed bony lines [3, 5, 6]. Translabial/transperineal ultrasound (TPUS) uses a transverse line along the inferior border of pubic symphysis as a reference line for diagnosing POP in different compartments [7–9]. On the other hand, the reference plane of the hymen, which is used for clinical examination, is a soft-tissue plane, which moves with the movement of the pelvic floor. The findings of clinical examination and imaging techniques may or may not correlate with each other or with the patient’s symptoms [10–12]. The variation in landmarks used for reference lines also means that different methods of imaging are not comparable. Imaging, however, can be used to understand how POP and associated symptoms interact. As an example, it is commonly used to assess anorectal symptoms, especially bowel evacuation disorders. The dilation and anterior ballooning of the rectum seen on MRI may not cause descent of the posterior vaginal wall and POP by physical examination. Indeed, the term “rectocele,” which is used to describe this MRI finding, is also commonly used to describe posterior vaginal wall prolapse. This often leads to confusion in the diagnosis and management of the conditions by different specialties. Clinical examination can visualize the vaginal wall descent, but it might be difficult to assess the visceral involvement [13]. Imaging techniques can identify the organs within the vaginal wall prolapse and hence improve the diagnostic accuracy of what the POP represents from an organ-based pathology. For example, it can help to differentiate the small bowel versus rectal descent in the settings of the clinically diagnosed posterior vaginal wall prolapse. The stage of POP may vary in the sitting up or standing position [12]. The non-invasive nature of imaging and convenience of assessment in a weight-bearing position are additional advantages of imaging for POP [3, 5, 6, 14]. Another question that arises is whether clinical examination or imaging might be more efficient in diagnosing POP of a particular compartment [15–17]. For example, a prolapse of the upper vagina, which may not be seen easily on clinical examination, may be better diagnosed using imaging techniques [18]. On the other hand, clinical examination may diagnose POP more accurately than imaging alone, and, more importantly, physical examination has been shown to correlate with POP symptoms [4, 17].

**Fig. 1:**
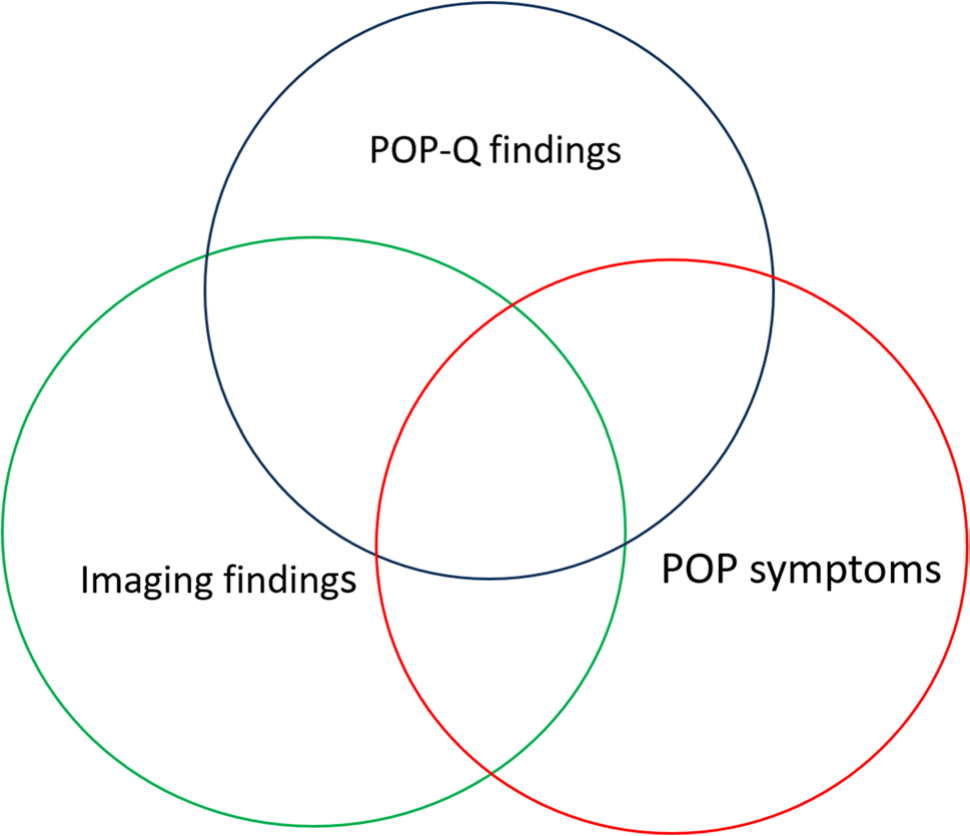
Venn diagram of pelvic organ prolapse (POP) physical findings, symptoms, and imaging findings. *POP-Q* Pelvic Organ Prolapse Quantification

The value of any diagnostic testing is traditionally assessed by non-experimental cross-sectional or cohort studies, which compare a test’s classification of a diagnosis with that of a reference standard. The conceptual starting point of a diagnostic test study is to apply the reference (or gold) standard to determine which study participants have the prolapse and which participants do not. In the case of prolapse, the Pelvic Organ Prolapse Quantification (POP-Q) examination is considered the gold standard among the urogynecology scientific community. However, although this view is widely accepted, it is not universally agreed upon. For example, in the colorectal literature, some studies call for other imaging modalities as a gold standard in assessing POP [19, 20]. The diagnosis of POP is further complicated by the fact that not all prolapse diagnosed by POP-Q is bothersome. Typically, the presence of symptoms is required to identify prolapse as clinically significant. Therefore, diagnostic studies should consider the fact that not all forms of prolapse identified on physical examination or imaging are symptomatic (Fig. 1).

A well-designed POP imaging accuracy study will need to include a clear definition of prolapse by POP-Q and symptoms, set up clear definitions of radiological findings identified as positive, calculate sensitivity and specificity, and ideally report likelihood ratio and receiver-operating curves (ROCs), which will allow the derivation of evidence-based cut-offs for this particular diagnostic modality. The area under the curve (AUC) on ROCs defines the accuracy of the test: the closer the AUC approaches 1, the more discriminatory value the test carries in distinguishing prolapse from normal controls. This chapter reviews different imaging techniques available for assessing POP and compares them with the clinical examination findings using a clinical diagnosis of prolapse according to either the POP-Q system or the Baden–Walker (BW) grading system. The BW half-way system preceded POP-Q and consisted of four grades: grade 0, no prolapse; grade 1, halfway to the hymen; grade 2, to the hymen; grade 3: halfway past the hymen; grade 4, maximum descent. It was included in the review to avoid exclusion bias, as the colorectal literature was late to adopt POP-Q and continued using the BW system long after it was introduced in 1994 into the urogynecology community.

## Materials and Methods

This manuscript is a narrative review. Nine international urogynecology experts in radiographic imaging in POP were assembled. The chair of the writing group was selected by the IUC chairs, the IUC steering committee with input from the IUGA Executive Committee. A competitive application process and invitation were developed for the other members (authors) of the writing group.

To complete an in-depth literature search on this topic, the authors assembled the search terms that they found most relevant to the imaging of POP. This list of terms was presented at the IUGA annual scientific meeting in 2020 for input from the membership. The additions from membership input made at that meeting were incorporated in the final search terms presented in Table 1. Regular meetings allowed for the group to collaborate on the outline and layout components of this narrative review. The PubMed, OVID Medline, and Scopus Databases were queried for the search terms noted in Table 1, between January 1990 and July 2020. The initial search, performed on 29 July 2020, produced 2,961 unique references. The references were uploaded into Covidence software and divided among the authors for initial screening. Each reference underwent an inclusion or exclusion criteria assessment by two independent reviewers (writing group members), with a third reviewer as a referee for tie-breaking inconsistencies. The Joanna Briggs Institute (JBI) scoping review guidelines were followed.

**Table 1:** MeSH search terms

Section 1	Introduction	ultrasound, pelvic organ prolapse imaging, fluoroscopy, pelvic floor disorders, pelvic floor imaging, mri pelvic floor, vaginal prolapse, rectocele, cystocele, defecography, proctography, radiology pelvic prolapse
Section 2	Role of Imaging in Prolapse	ultrasound, pelvic organ prolapse imaging, fluoroscopy, pelvic floor disorders, pelvic floor imaging, mri pelvic floor, vaginal prolapse, rectocele, cystocele, defecography, proctography, radiology pelvic prolapse
Section 3	MRI—Technique and Evaluation of Prolapse	mri pelvic prolapse, magnetic resonance imaging prolapse, mri pelvic floor, mri pelvic laxity, mri pelvic relaxation, mri cystocele, mri rectocele, mri anterior compartment, mri posterior compartment
Section 4	Role of X-ray and CT scan	x-ray abdomen, barium, computed tomography pelvis, CT scan pelvis, CT scan uterovaginal prolapse, radiologic imaging prolapse, radiographic imaging pelvis, fluoroscopy pelvis, contrast imaging, cysto-urethrography
Section 5	Ultrasound—Overview of Techniques: Transperineal, Introital, Transvaginal	transperineal ultrasound, translabial ultrasound, transvaginal ultrasound, pelvic prolapse, three dimensional ultrasound pelvic floor, 3 D ultrasound, 4 D ultrasound pelvic floor, introital ultrasound
Section 6	Ultrasound Anterior Compartment	ultrasound bladder, ultrasound urethra, bladder imaging, ultrasound anterior compartment, ultrasound cystocele, urethrocele, anterior vaginal defect, levator hiatus
Section 7	Ultrasound Middle Compartment	uterine prolapse, vaginal vault prolapse, levator hiatus, levator ani muscle, posthysterectomy prolapse, ultrasound vaginal prolapse, ultrasound uterovaginal prolapse, ultrasound vaginal vault prolapse, apical prolapse, cervical prolapse, ultrasound genital hiatus, levator ballooning
Section 8	Ultrasound Posterior Compartment	transperineal ultrasound, introital ultrasound, rectocele, ultrasound posterior compartment, levator ani imaging, ultrasound levator hiatus, enterocele, rectocele, posterior vaginal defect, perineal hypermobility

**Fig. 2:**
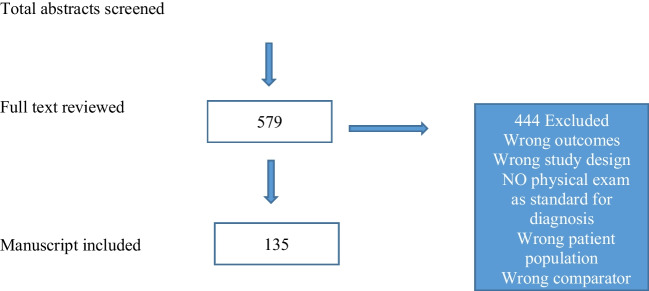
Preferred Reporting Items for Systematic Reviews and Meta-Analyses (PRISM) diagram of the studies reviewed

Following the initial review, all abstracts were reviewed by two reviewers independently, and conflicts were resolved by a third team member, with the aim of eliminating the studies where the primary focus of imaging use was not the diagnosis of POP and where physical examination (POP-Q or other prolapse grading system) was not used as a gold-standard reference for POP diagnosis. This process resulted in 581 manuscripts relevant to the goal of the narrative review. The full-text manuscript reviews were performed by two reviewers independently rated for inclusion or exclusion, according to the JBI checklist. The final inclusion list consisted of 112 manuscripts and was made as a consensus discussion among all reviewers. Figure 2 shows the Preferred Reporting Items for Systematic Reviews and Meta-Analyses diagram of the article selection process.

Next, the data extraction from the manuscripts was performed, using a standardized data extraction sheet developed specifically for this project. The data collected included study geographic location, study design, number of participants, relevant imaging technique details, types of reference lines used (if any), prolapse compartment (anterior, apical, posterior), and testing validation methods. The sections were divided into different imaging techniques and included X-ray/fluoroscopy, CT scan, MRI, and ultrasound. At least two team members contributed to the data synthesis of each section.

The writing group members produced versions of the manuscript incorporating the edits provided by all members until a final first draft was achieved. This was then circulated to several chosen referees before undergoing peer review. The IUC peer review process involved four rounds of review, including review by the IUC co-chairs, the IUC steering committee members, the IUGA general membership (through an online process), and finally the IUGA board members. The manuscript was then submitted for peer review to the *International Urogynecology Journal*.

## Results

### X-Ray/Fluoroscopy

A total of 4 cohort studies met the inclusion criteria. Three studies used the POP-Q system for the diagnosis of prolapse [13, 21, 22] and one study used the BW grading system [23]. Three studies focused on posterior wall compartment prolapse (defecography) [13, 22, 23] and one addressed the anterior compartment [21].

#### Variation in Technique

**Fig. 3:**
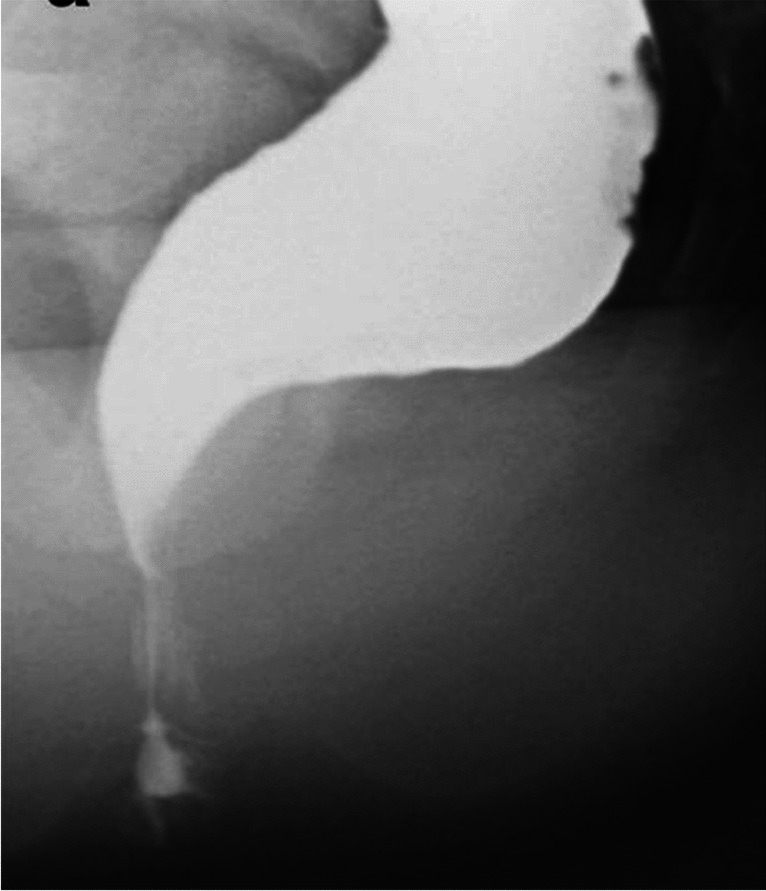
Normal fluoroscopy with rectal barium opacification

All studies were performed in the sitting position on a commode, with maximum straining, squeezing, and at rest (Fig. 3). The biggest variation in the technique was evident in the methods used for opacification of the rectum, vagina, bladder. and small bowel. For posterior prolapse imaging, Altman et al. used oral barium contrast medium, intraperitoneal and intravesical omnipaque solution, and barium paste contrast medium in the vagina and the rectum during defecoproctography (DCP) [13]. Finco et al. also used barium paste opacification of the rectum and vagina but added barium paste to the perianal skin, and used iothalamic acid for bladder opacification [23]. Groenendijk et al. limited opacification to the small bowel and rectum by using barium sulfate suspension meal and barium enema [22]. The only study reporting on anterior compartment prolapse described the extensive opacification technique involving intraperitoneal and intravesical omnipaque administration in addition to vaginal and rectal barium paste [21].

#### Definition of Cases and Controls and Radiographic Markers

Three studies reported on symptoms associated with prolapse but did not use those symptoms in defining clinically significant prolapse. The clear clinical definition of prolapse was used only in one study: Groenendijk et al. defined clinically significant prolapse of the posterior wall as ≥ POP-Q stage II [22]. The clear definition of an abnormal radiographic finding in the anterior compartment was reported by Altman et al., describing the descent of opacified urinary bladder below the pubococcygeal line as abnormal [21]. Studies focusing on the posterior compartment used definitions of abnormal radiographic findings describing the apex of the rectocele as a common reference point. Groenendijk et al. measured the distance from the rectocele apex to the expected rectal lining of the anterior rectal wall [22]. Finco et al. and Altman et al. measured the distance between the rectocele apex and the line extended through the anal canal axis [13, 23]. In addition, Finco et al. classified radiographic findings of rectocele as grades I, II, and III, using this distance, and defined radiographic rectocele as grade I when it was less than 2 cm long, grade II for 2–4 cm, and grade III when it was over 4 cm [23].

#### Diagnostic Accuracy Reporting

There were no studies clearly reporting on the sensitivity or specificity of fluoroscopic testing, in relationship to clinical examination. Studies examined only the correlation between clinical and radiological findings. There was a moderate correlation between the clinical and radiological diagnosis of anterior wall prolapse (degree of correlation *r* = 0.67) [13]. There was poor correlation between fluoroscopic imaging of the posterior compartment and clinical examination for posterior vaginal prolapse (degree of correlation *r* = 0.49) [21]. Although increasing the size of the rectocele on defecography moderately correlated with difficulty in rectal emptying (*r* = 0.59), there were no other significant associations between symptoms and anatomical findings on imaging [23].

### Computerized Tomography

Only one study was identified reporting on the diagnostic value of computerized tomography (CT) [24]. The study included only seven patients and commented on all compartments. The authors did not use POP-Q as the gold standard for POP diagnosis but rather identified the presence or absence of prolapse in specific compartments during surgical correction as an ultimate reference point. They used extension of the bladder base past the PCL as a radiographic marker for anterior prolapse, and the distance between the line from the anterior margin of the anal canal and the anterior wall of the rectum greater than 2 cm as the radiographic definition of posterior prolapse. CT findings were false negative for all three sites of prolapse in one patient. There were no false-positive cases on CT, when compared with surgical findings.

Because of the small number of studies identified using search terms specific to fluoroscopic and CT imaging of prolapse, the original search was extended past January 2000 to include manuscripts published as early as January 1990. The extended search added no additional manuscripts for CT and one additional manuscript in fluoroscopy [25]. Brubaker et al. evaluated 30 women with prolapse beyond introitus straining in a sitting position with oral contrast medium, and vaginal, rectal, and bladder opacification [25]. The specific radiographic findings consistent with prolapse were not clearly defined and cystocele and rectoceles were reported as present or absent. Radiographic markers were described as heterogeneous with comments on their appearance such as “hour glass shaped.” The study did not report on the sensitivity or specificity of testing but the authors concluded that 11 patients had a modification of their surgical plan based on the information obtained from imaging.

#### Conclusion

There is no standardization in CT and fluoroscopic imaging techniques with regard to diagnosing POP. Opacification modalities vary greatly, and the definitions of radiographic findings consistent with prolapse are often unclear. There are no appropriately designed studies describing the diagnostic accuracy of fluoroscopy or CT in the diagnosis of POP. The summary of studies is presented in Table 2.

**Table 2:** Computerized tomography (CT) and fluoroscopy

Reference	Technique	Physical examination	POP symptoms considered in the analysis	Main reported outcome
Altman et al. [13]	Cystodefecoperitoneography, video	BW	UDI, DDI	History of pelvic surgery, size of prolapse of the posterior vaginal wall, and the presence of constipation (assessed by a questionnaire) are predictors of the presence of abnormal defecography
Altman et al. [21]	Cystodefecoperitoneography, video	BW	None	Moderate correlation between clinical and radiological findings in patients with anterior vaginal wall prolapse. New definition of cystocele with lead markers at the introitus did not improve the correlations
Finco et al. [23]	Colpocystodefecography	BW	KESS	Proportions of patients diagnosed with rectocele radiographically and with BW did not differ before surgical intervention, but they did differ after surgery for POP
Groenendijk et al. [22]	Defecography	POP-Q	DDI, UDI, CRADI	Two groups with rectocele (stage II and higher and stage I and lower) were compared. Symptoms were compared in groups defined by PE and by defecography. No relation was found between bowel complaints and posterior wall prolapse evaluated by clinical examination (*p* = 0.33), nor between bowel complaints and rectocele (*p* = 0.19) assessed by defecography
Brubaker et al. [25]	Dynamic fluoroscopy	Physical examination	None	Dynamic fluoroscopy improved pre-surgical evaluation by identifying enterocele in 26 out of 30 patients
Pannu et al. [24]	CT	Surgical exploration	None	CT findings were false negative for all three sites of prolapse in one patient. There were no false-positive cases on CT when compared with surgical findings

*BW* Baden–Walker, *POP-Q* Pelvic Organ Prolapse Quantification, *UDI* Urinary Distress Inventory, *DDI* Defecatory Distress Inventory, *KESS* Knowles Eccersley Scott Symptom Score, *CRADI* ColoRectal-Anal Distress Inventory, *POP* pelvic organ prolapse, *PE* physical examination, *CT* computerized tomography

### Magnetic Resonance Imaging

A total of 25 studies met the inclusion criteria for assessing prolapse via MRI. Most studies were cohort cross-sectional, with 5 out of 19 describing cohorts of patients planning surgical intervention for prolapse. All but two studies [18, 26] used POP-Q for describing patient prolapse type and severity.

#### Variation in Technique

**Fig. 4:**
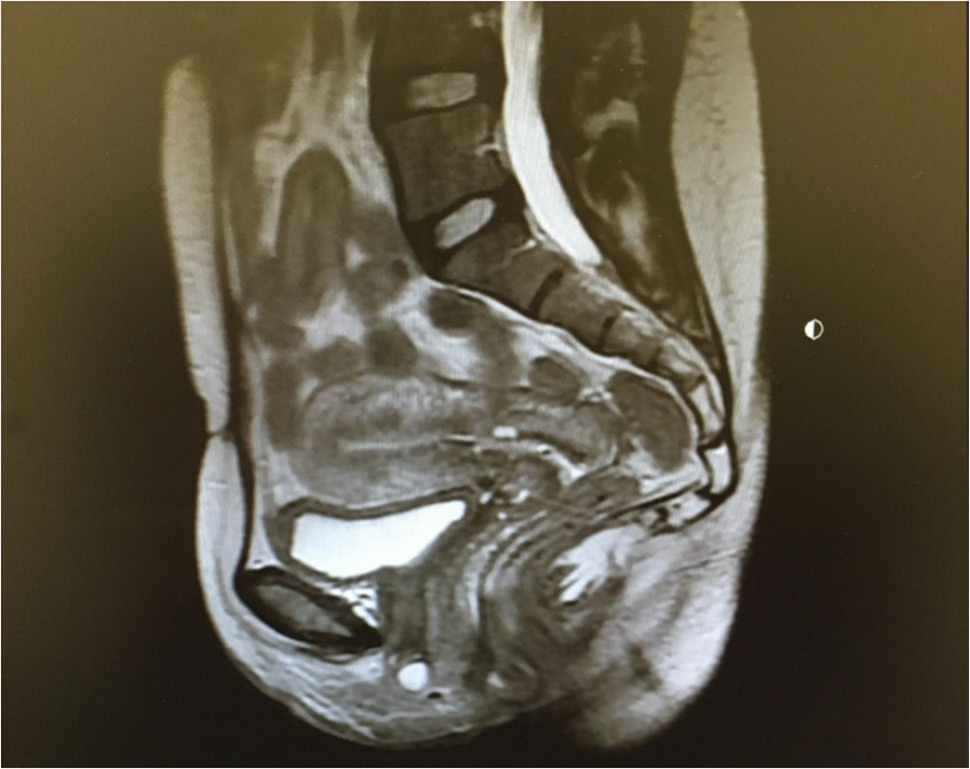
Magnetic resonance image at rest with no prolapse

**Fig. 5:**
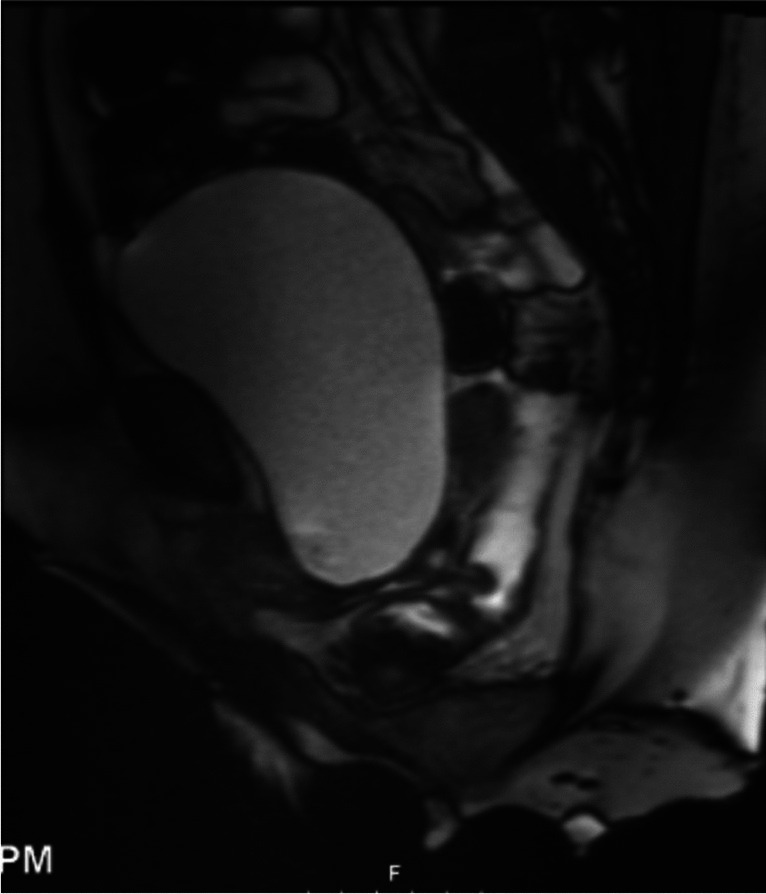
Magnetic resonance image demonstrating a posterior defect with enterocele containing small bowel and small bowel mesentery

With the exception of one study, all studies used a T2-weighted basic pulse sequence, which enhances the signal of water (Figs. 4, 5) [26]. The strength of a magnetic field in an MRI machine varied from 0.25 to 3 Tesla with approximately half of studies reporting on the 1.5-Tesla MRI technique. All studies were performed in the supine position and images obtained at rest and during straining. Some studies added images obtained during squeezing and contraction of the pelvic floor muscles [6, 17, 27]. One study examined patients in the supine position during defecation [18]. Delaney et al. hypothesized that prolapse in one vaginal wall can be obscured by a competing defect in the opposite vaginal wall in cases of multicompartmental prolapse. The authors examined the effect of the reduction of the opposing vaginal wall with the vaginal speculum blade and concluded that in cases of advanced POP, the speculum pressing onto the most dependent portion of the vaginal wall prolapse reveals additional prolapse in the opposing compartment in 59% of the patients [28]. Abdulaziz et al. evaluated the effect of positioning (standing, sitting, and supine) on the diagnostic accuracy of MRI in POP quantification and concluded that the maximal extent of prolapse is best evaluated in the standing position [29]. Tumbarello et al. established that 95% of women extended their prolapse further in the supine position with repetitive Valsalva maneuvers [30]. About half of the studies used vaginal and/or rectal gel to enhance opacification and one study was specifically aimed at assessing the effect of the addition of vaginal and rectal gel on POP MRI imaging by comparing opacified and non-opacified imaging techniques [31]. Oral contrast medium was used only in the study with T1-weighted images [26]. One study reported on the use of intramuscular butylscopolamine to reduce intestinal mobility [32] and one study described gadolinium solution infused into the bladder in addition to using vaginal and rectal gel. [33]

#### Definition of Cases and Controls and Radiographic Markers

Only four studies collected data on the symptoms of prolapse and used validated questionnaires [32, 34–36]. The most commonly used questionnaires were the Urinary Distress Inventory (UDI), the Defecatory Distress Inventory (DDI), the Incontinence Impact Questionnaire (IIQ), and the ColoRectal-Anal Distress Inventory. One study included correlation of prolapse and symptoms, but did not use a validated questionnaire [37]. All but one of the studies did not utilize questionnaires in defining POP as symptomatic or clinically significant [36]. The biggest variation existed in definitions of MRI findings: multiple midsagittal pelvic reference lines were described to quantify prolapse using MRI (Table 3). An attempt was made to standardize MRI lines, by introducing the sacrococcygeal–inferior pubic point line; however, this proposed reference line was not universally accepted. Subsequently, radiographic definitions of prolapse in reference to multiple lines varied greatly. Largely, studies reported either on distances between the pre-determined or leading portions of the prolapsing organ and the selected reference line or on different radiographic stages of prolapse using arbitrary cut-off values (Table 4). Xie et al. introduced the term “exposed vaginal length,” measured from the point where the posterior vaginal wall separates from the anterior wall to the ventral tip of the perineal body, as a potential tool to diagnose posterior compartment prolapse [36]. Rodrigues Jr et al. explored the value of estimated levator ani volume (LASV) in prolapse staging and found that LASV can be estimated using MRI and shows good correlation with 3D images on MRI; the clinical relevance of this finding needs to be studied [38]. Lammers et al. used pubovisceral muscle avulsions on MRI to correlate with prolapse in different compartments and found that pubovisceral avulsions, presence, and severity correlated with signs and symptoms of prolapse. [32]

**Table 3:** Magnetic resonance imaging reference lines

Line	Definition	References
Pubococcygeal line	Extending from the inferior-most portion of the symphysis pubis to the tangent of the last coccygeal joint. Points of interest are measured as a vertical distance to the reference line corresponding to the levator muscles	Abdulaziz et al. [29], Agildere et al. [26], Broekhuis et al. [17], Delaney et al. [28], Etlik et al. [14], Grob et al. [3], Hodroff et al. [33], Lakeman et al. [34], Lin et al. [18], Pannu et al. [31], Pollock et al. [68], Siegmann et al. [37], Singh et al. [61], Torricelli et al. [62]
Midpubic line	Drawn across the midsagittal aspect of the pubic bone through the approximate level of the vaginal hymen corresponding to the level of the hymen	Abdulaziz et al. [29], Barakat et al. [6], Cortes et al. [63], Fauconnier et al. [64], Lakeman et al. [34], Pannu [31], Singh et al. [61], Woodfield et al. [65], Xie et al. [36]
Horizontal line	Measures the width of the pelvic floor hiatus in the anteroposterior dimension. Measured from the inferior tip of the pubic symphysis to the posterior circular fibers of the anorectal junction	Xie et al. [36], Lin et al. [18], Lakeman et al. [34], Gupta et al. [16], Broekhuis et al. [17], Abdulaziz et al. [29]
Mid-anal line	Line extending through the middle of the anal canal in a resting position of the anorectal junction 2 cm above the plane of the ischial tuberosities	Xie et al. [36], Lin et al. [18], Abdulaziz et al. [29], Sayed et al. [66]
Internal anal sphincter line	Reference line placed through the ventral aspect of the internal anal sphincter	Xie et al. [36]
Hiatus line	Measures the distance from the pubis to the posterior anal canal	Xie et al. [36], Comiter et al. [67]
Perineal line	Reference line from the inside of the pubic symphysis to the front tip of the perineal body	Xie et al. [36], Lakeman et al. [34], Fauconnier et al. [64], Abdulaziz et al. [29]

**Table 4:** Radiographic definitions of prolapse

Radiographic distance to the reference line	Reference	Radiographic staging of POP	Reference
Difference between the coordinates of maximal prolapse for anterior and posterior vaginal wall and apex and reference line	Barakat et al. [6], Fauconnier et al. [64], Lakeman et al. [34], Tumbarello et al. [30], Xie et al. [36]	Stages of prolapse ranging from I to IV as referenced by the distance from the mid-pubic line: stage I MPL < −1 cm (less than 2-cm descent); stage II between −1 and + 1 distance from the MPL (between 2- and 4-cm descent); stage III more than + 1 cm from the MPL but less than + (TVL-2) (more than 4-cm descent), stage IV more than + (TVL-2) or more than 4-cm descent	Cortes et al. [63]
In the anterior compartment, the posterocaudal-most point of the anterior vaginal wall was used; in the central compartment, the distal-most point of the cervix or the vaginal vault; and in the posterior compartment, the anterocaudal-most point of the posterior vaginal wall using reference	Broekhuis et al. [17]	Modified Sigh et al.: 1st 0.5–2 cm movement of the pelvic organs during straining, 2nd 2–4 cm movement, but not below the PCL, 3rd > 4 cm movement or below the PCL, 4th > 10 cm movement below the PCL	Etlik et al. [14]
The positions of the bladder neck, bladder base, and uterine cervix (or vaginal apex) were recorded as positive if above and negative if under the PCL	Delaney et al. [28]	HMO classification for MRI grading of pelvic organ prolapse: 0 above the H line; 1, 0–2 cm below the H line; 2, 2–4 cm below the H line; 3, more than 4 cm below the H line	Gupta et al. [16]
Distances to the anterior part of the cervix, bladder neck, and pouch of Douglas were measured to the PCL	Grob et al. [3]	Stage I: all organs lying above the MPL; stage II: pelvic organs lying < 1 cm proximal to or distal from the MPL; stage III: distal-most portion of the prolapse is > 1 cm below the MPL but protrudes no further than 2 cm less than the total vaginal length; stage IV: complete eversion	Singh et al. [61]
Measures bladder neck height or the perpendicular distance from the bladder neck to the reference line, the posterior urethrovesical angle, and the posterior levator plate angle relative to the PCL	Hodroff et al. [33]	Mild: organ descended < 3 cm below the PCL, moderate: between 3 and 6 cm below the PCL; severe: if more than 6 cm below the PCL. Rectocele was classified as mild if protruding less than 2 cm past the line tangential to the anterior wall of the anal canal, moderate if protruding 2–4 cm, and severe if protruding more than 4 cm	Torricelli et al. [62]
Cystocele: any portion of the bladder herniated below the PCL. Rectocele anterior bulging (> 1 cm) of the anterior rectal wall compared with static imaging	Lin et al. [18]	Anterior compartment, the reference point was the posterior- and inferior-most aspect of the bladder base. In the apical compartment, the reference point was the anterior cervical lip or the posterior superior vaginal apex if the woman was post-hysterectomy. In the posterior compartment, the anterior aspect of the anorectal junction served as the point of reference. Organ prolapse < 3 cm below the PCL small, 3 to 6 cm below moderate, and > 6 cm below the PCL large prolapse	Woodfield et al. [65]
Bladder: cystocele defined as 1) below the PCL, 2) below the MPL or < = 3 cm above the MPL; apical POP: 1) below the PCL, 2) below the MPL or < = 5 cm above the MPL; small bowel: 1) below the apical third of the vagina; rectum: more than 3-cm anterior bulge relative to the anal canal	Pannu et al. [31]	No staging system proposed	
Organ prolapse (O line) was used to categorize cystocele, when a portion of the bladder prolapsed below the PCL. Apical prolapse was measured with respect to the PCL. Rectocele was defined as anterior bulging > 1 cm of the anterior wall of the rectum compared with static imaging	Pollock et al. [68]	No staging system proposed	
Cystocele was diagnosed if the bladder base descended below the PCL on straining. Rectocele was present if the anterior rectal wall was pouching out ≥ 2 cm at defecation	Siegmann et al. [37]	No staging system proposed	
Anterior compartment—the distal-most part of the bladder. Apical compartment—the leading edge of the vaginal cuff or the location of the cervix. Posterior compartment—the anorectal junction in relation to the PCL	Van der Weiden et al. [35]	No staging system proposed	

*PCL* pubo-coccygeal line, *MPL* midpubic line, *POP* pelvic organ prolapse, *TVL* total vaginal length, *HMO* H line, M line, organ prolapse, *MRI* magnetic resonance imaging

#### Diagnostic Accuracy Reporting

The majority of the studies reported on the association or correlation of prolapse with physical examination findings but did not have sensitivity or specificity calculated or ROC reported. Findings of advanced prolapse stages appear to correlate better with MRI POP diagnosis than POP-Q stages I and II. The correlation of POP-Q prolapse diagnosis is slightly better in the anterior compartment than in the apical and posterior compartments. Only one study reported ROCs for different radiographic markers assessing posterior compartment prolapse [36]. The study compared the diagnostic value of eight existing reference lines and a new parameter, the “exposed vaginal length,” in the diagnosis of posterior compartment prolapse. The study focused on the ability of MRI to detect the size and not the POP-Q stage of prolapse, as the authors believed that POP-Q is not designed to assess the prolapse size, which is the parameter that the authors felt most consistently correlated with bothersome symptoms. The exposed vaginal length outperformed the traditional reference lines in diagnosing prolapse size, with an AUC of 0.95. This measurement can discriminate large posterior compartment prolapse from small, with a cut-off value of 2.9 cm. The “perineal line-internal pubis” showed the highest sensitivity and specificity among traditional lines, with an AUC of 0.91 [36].

#### Conclusion

Magnetic resonance imaging findings appear to correlate somewhat better with POP-Q staging in the anterior compartment and in more advanced stages of prolapse. The lack of standardized definitions for reference lines and a lack of reporting on test accuracy made it difficult to compare study results.

### Ultrasound

Out of the 50 studies that met the inclusion criteria for assessing POP via ultrasound, 44 explored the perineal ultrasound technique, consistent with AIUM/IUGA practice guidelines [39]. The remainder of the studies focused on endovaginal, endoanal, and trans-abdominal ultrasound.

#### Transperineal Ultrasound

The vast majority of the TPUS studies were cohort cross-sectional, with only 10 studies designed as case–control cross-sectional. Two studies used the BW or the Green classification of cystoceles, with the remainder reporting POP according to the POP-Q. The Green classification of cystocele takes into account the urethrovesical angle and the level of urethral involvement in anterior vaginal wall descent [40]. Three studies included patients planning surgery for prolapse.

##### Variation in Technique

**Fig. 6:**
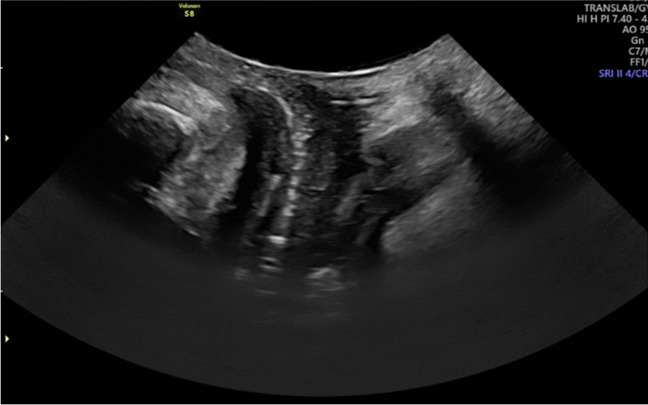
Transperineal ultrasound at rest

**Fig. 7:**
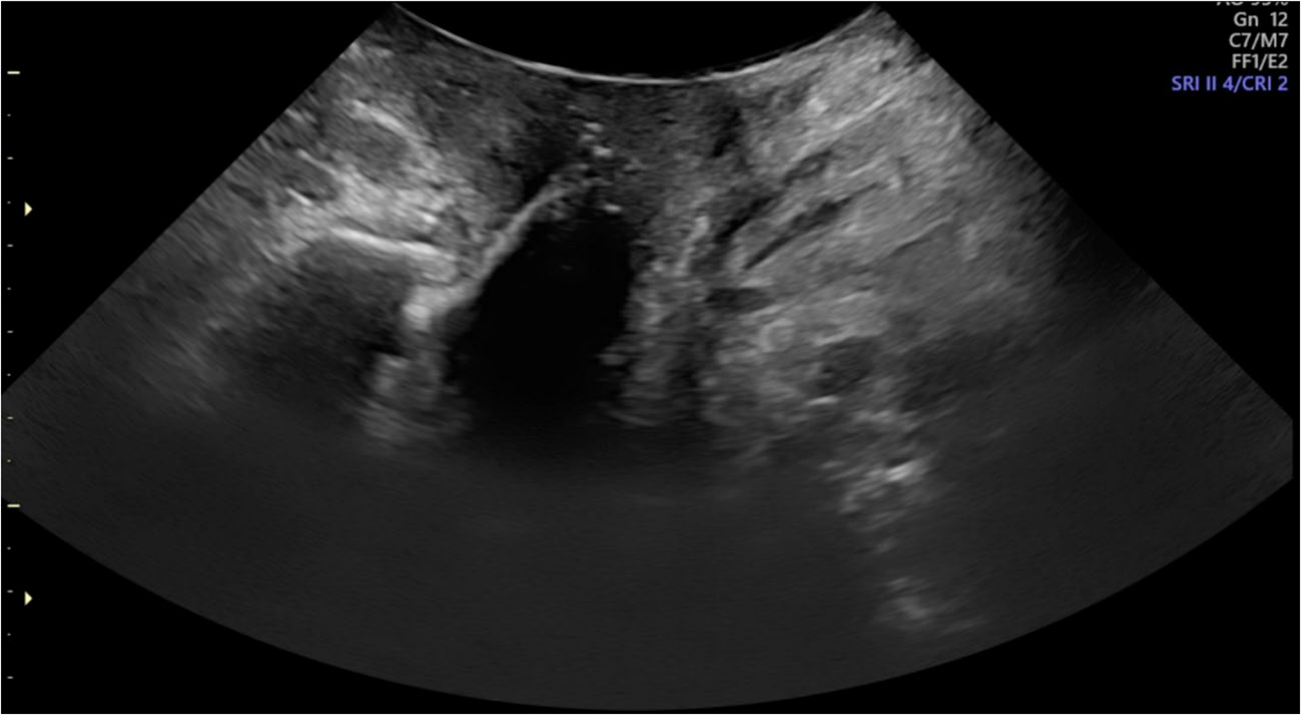
Transperineal ultrasound at rest with Valsalva demonstrating anterior compartment prolapse

Technique variation in transperineal ultrasound was minimal in the studies published after 2004 following a standardized protocol popularized by Dietz et al. (Figs. 6, 7) [41, 42]. Most of the studies were performed in supine position with no organ opacification. The transducer was applied to the perineum lightly placed to minimize pressure so as not to reduce maximal descent. The technique’s sensitivity to positional changes was examined by Rodriguez-Mias et al. who studied the effect of standing position on US accuracy, to assess if established diagnostic cut-offs for POP need to be changed. The authors concluded that parameters describing organ descent are not affected by the standing position but hiatal diameters change enough to consider a new cut-off [43]. Braverman et al. demonstrated that diagnostic performance of sonographic markers predicting prolapse is only marginally better in standing position [12].

##### Definition of Cases and Controls and Radiographic Markers

Two thirds of the studies included assessments of symptoms, but only five used validated questionnaires. The most commonly used questionnaire was the Pelvic Floor Distress Inventory (PFDI-20) [44]. Most of the studies did not use symptoms or validated questionnaires in the definition of clinically significant prolapse, but rather explored the association between ultrasound diagnosis of POP and the presence of clinical symptoms, without quantifying severity. However, several studies focused on the diagnostic accuracy of ultrasound in relationship to POP symptoms, rather than the POP-Q, and attempted to define cut-offs for significant pelvic organ descent on the basis of prolapse symptoms [7]. Ten studies used the definition of POP-Q stage II as clinically significant. There were two main categories of sonographic markers utilized to predict POP: the measurements of descent describing organ position relative to the inferior margin of the symphysis pubis and measurements relative to the levator plate (Table 5). The most frequently quoted topographic organ descent cut-offs were ≥ 10 mm and ≥ 15 mm below the symphysis pubis for the bladder and rectal ampulla [7]. In studies focusing on uterine prolapse, the clinical definition of prolapse was defined as descent of the cervix to 15 mm above the symphysis pubis or lower. The highest variation was amongst the cut-off values describing the apical compartment. They varied between 15 mm above the symphysis pubis to 0 mm (symphysis pubis level). However, some studies explored other sonographic markers such as tenting of the paravaginal fornices in the axial plane (presumably describing a paravaginal defect) [45], discontinuity in the anterior anorectal muscularis that resulted in a diverticulum of the rectal ampulla extending into the vagina (“true rectocele”) [46], vaginal canal shape described as H, U, and eye shaped [47] or morphology and axial orientation variations of the levator plate [48].

**Table 5:** Sonographic markers for pelvic organ prolapse

Topographic imaging of organ descent	References	Measurements of the levator plate	References
Anterior: bladder descent below the symphysis	Xuan et al. [69], Lone et al. [4, 70], Dietz et al. [8, 11, 71], Kluivers et al. [72], Lai et al. [73], Rodriguez-Mias et al. [43], Wen et al. [53], Bu et al. [74], Chantarasorn and Dietz [9], Najjari et al. [75], Schettino et al. [76], Barakat et al. [6], Braverman et al. [12], Broekhuis et al. [17], Zhu et al. [77]	Levator muscle avulsion]	Trutnovsky et al. [78], Dietz and Beer-Gabel [79], Lai et al. [73], Pattillo Garnham et al. [80], Ying et al. [48], Zhuang et al. [50], Huang et al. [60], Zhu et al. [77]
Posterior: the anterocaudal-most point of the anterior rectal wall below the symphysis	Xuan et al. [69], Volloyhaug et al. [15], Lone et al. [4, 70], Dietz et al. [11, 71, 81], Dietz and Steensma [82], Dietz and Lekskulchai [7] Dietz and Korda [83], Barakat et al. [6], Rodrigues et al. [38], Kluivers et al. [84], Lai et al. [73], Rodriguez-Mias et al. [43], Wen et al. [53], Braverman et al. [12], Broekhuis et al. [17], Zhu et al. [77]	Levator diameter (vertical and horizontal)	Majida et al. [85], Kozma et al. [86], Dietz and Beer-Gabel [79], Pineda et al. [87], Huang et al. [60], Wen and Zhou [51], Ying et al. [48], Zhuang et al. [50], Wen et al. [88]
Apical: uterine descent in relation to the symphysis	Xuan et al. [69], Volloyhaug et al. [15], Lone et al. [4, 70], Dietz et al. [8, 11, 71], Kluivers et al. [84], Lai et al. [73], Rodriguez-Mias et al. [43], Wen et al. [47, 53], Wu et al. [49], Barakat et al. [6], Braverman et al. [12], Broekhuis et al. [17], Shekand Dietz [89], Zhu et al. [77]	Levator hiatal area (also described as levator ballooning)	Xuan et al. [69], Abdool et al. [90], Athanasiou et al. [57], Dietz and Beer-Gabel [79], Lai et al. [73], Pattillo Garnham et al. [80], Rodriguez-Mias et al. [43], Speksnijder et al. [91], Zhuang et al. [50]

##### Diagnostic Accuracy Reporting

Fifteen out of 44 studies commenting on the value of transperineal ultrasound in the diagnosis of POP reported measures of diagnostic test accuracy (area under the curve for ROC analysis). Five studies reported on topographic sonographic markers and eight focused on the measures of the levator plate, with two studies exploring both measures. Although the sonographic definitions were fairly consistent across the literature, the studies used heterogeneous definitions of prolapse as reference standards. Some studies focused on detecting the symptoms of prolapse and some attempted to report on diagnostic test discrimination between POP-Q stages. Others defined clinically significant prolapse as ≥  POP-Q stage II (Table 6). The definition of clinically significant prolapse by POP-Q varied by compartment, with the majority of studies making the distinction for ≥  POP-Q stage II for anterior and posterior compartments and ≥ POP-Q stage I for the apical compartment. None of the studies used validated questionnaires to define clinically significant POP. All studies were cohort cross-sectional by design and included a large number of participants, but were limited to two populations: predominantly white in Australia and Asian in China. AUCs ranged from 0.59 for an ultrasound topographic marker predicting uterine POP symptoms in the case of uterine prolapse, to 0.94 for hiatal area at Valsalva predicting POP-Q stage III. It is worth noting that an AUC of 0.5 carries no diagnostic value in detecting a pathological condition and indicates that the test is performing no better than flipping a coin.

**Table 6:** Accuracy reporting for transperineal ultrasound studies

Reference	Ultrasound marker	Details	Prolapse reference standard definition	ROC statistics
Trutnovsky et al. [78]	Levator plate	Scoring system for puborectalis avulsion: 6-point and 12-point systems	Symptoms and signs of POP in all compartments dichotomized as presence or absence	AUCs were 0.619 and 0.633 for the 6-point and the 12-point scales respectively. The discrete variable “avulsion” a cut-off value of “6” was chosen for the 12-point scale on the basis of these ROC curves. This resulted in 32.8% sensitivity and 86.0% specificity for predicting significant POP on ultrasound. The association between avulsion and physical findings of POP (POP-Q) was stronger than the association of avulsion with ultrasound findings of POP
Dietz et al. [81]	Topographic	Measurement of rectal descent relative to the symphysis, rectocele depth measurement	Posterior compartment prolapse associated with symptoms of vaginal digitation and incomplete bowel emptying	AUCs are 0.61 for detecting vaginal digitation and 0.614 for incomplete bowel emptying on the one hand and rectocele depth on the other. The cut-off depth of 15 mm provides sensitivities of 66% for vaginal digitation and 63% for incomplete emptying, and specificities of 52 and 57% respectively
Dietz and Lekskulchai [7]	Topographic	Measurement of rectocele and cystocele descent below the symphysis pubis	Dominant compartment POP associated with the feeling of a vaginal lump or bulge, or a dragging sensation	AUCs 0.857 vs 0.821 for anterior vs posterior compartment for predicting symptoms of POP
Kluivers et al. [72]	Topographic	Maximum descent of the leading edge of the bladder, the cervix or vaginal vault and rectum in millimeters	Symptomatic prolapse confirmed by POP-Q or BW and associated with the feeling of a vaginal lump or bulge, or a dragging sensation	AUCs indicating the probability of symptoms of prolapse with increasing stages, was 0.778 for the POP-Q, 0.783 for BW ordinal stages and 0.715 for ultrasound quantification. The cut-off point, with equal costs of misclassification, was at the hymen (0 cm) in the POP-Q, stage 2 in the ordinal stages and 14 mm below the reference line through the symphysis pubis for ultrasound
Lai et al. [73]	Levator plate	The depth of discontinuity between the insertion of the muscle and the rami pubis in the case of abnormal insertion of the puborectalis muscle. Measured in the plane of minimal hiatal dimension	POP symptoms were defined as a vaginal lump/bulge or a dragging sensation. Significant clinical prolapse was defined as POP stage II or higher	AUC 0.82 for POP stage II or higher, 0.84 for POP symptoms, 0.77 for significant POP on ultrasound, and 0.79 for hiatal ballooning. ROC showed that a cut-off depth for levator muscle injury of 7 mm yielded sensitivity of 62% and specificity of 80% for POP symptoms
Pineda et al. [87]	Levator plate	Midsagittal diameter of the hiatus on Valsalva	Significant clinical prolapse was defined as POP stage II or higher	AUC of 0.637 for AP diameter on Valsalva. AUC 0.71 for the relationship between midsagittal AP diameter and clinical findings of significant prolapse, 0.751 for significant prolapse diagnosed on ultrasound. A cut-off of 6 cm of the AP hiatal diameter on Valsalva yielded a specificity of 0.64 and a sensitivity of 0.7 for detecting significant prolapse on ultrasound
Rodriguez-Mias et al. [43]	Topographic and levator plate	Maximum descent of the leading edge of the bladder, the cervix or vaginal vault and rectum in millimeters in the standing position. Levator hiatus area in the standing position	Symptoms of POP with clinically significant POP as POP-Q stage II and above for the anterior and posterior compartments and POP-Q stage ≥ I for the apical compartment	AUCs 0.698 for cystocele, 0.593 for uterine descent, 0.635 for the rectal ampulla, and 0.706 for levator hiatal area respectively vs symptoms of POP. A hiatal area on Valsalva of 29 cm^2^ was found to be the optimal cut-off when imaging is performed in the standing position
Wen et al. [47]	Topographic	Maximal uterine descent and shape of the vaginal canal	Symptoms of POP with clinically significant POP as POP-Q stage II and above	The AUC was 0.69 for apical prolapse. The ROC curve proposed a cut-off of 10 mm above the symphysis pubis for uterine prolapse (POP stage ≥ I) with sensitivity of 74% and specificity of 64%. An eye-shaped vaginal canal with an AP diameter of greater than 10 mm in the rendered axial plane was a sign of uterine prolapse
Wen et al. [53]	Measures of levator plate	Hiatal area, AP diameter of hiatal area and width of hiatal area measured in centimeters at rest, on Valsalva maneuver, and on pelvic floor muscle contraction. All measures transformed in Z-score. Z-score 5 (measured value – predicted mean value)/predicted standard deviation	POP-Q stage II or higher defined as symptomatic prolapse. Symptomatic POP is defined as subjective symptoms of the bulge. Substantial POP on translabial ultrasound defined as a cystocele to at least 10 mm below the symphysis, uterine descent to 15 mm above the symphysis or lower, rectal ampulla/enterocele descent to at least 15 mm below the symphysis, or a combination thereof	AUCs of 0.69, 0.87, and 0.86 for Z-scores for POP-Q stage II or higher substantiate POP on translabial ultrasound, and symptomatic POP. The levator hiatal area cut-off was 20 cm^2^ with sensitivity of 81% and specificity of 58% for POP-Q stage II or higher, sensitivity of 86% and specificity of 70% for symptomatic POP, and sensitivity of 87% and specificity of 73% for substantiate POP on translabial ultrasound. ROC analysis illustrated that no diagnostic cut-off value can be used to distinguish the normal state and POP stage I. Patients with POP stage I were always clinically asymptomatic
Wen et al. [52]	Measures of levator plate	Hiatal area, AP diameter of the hiatal area and width of the hiatal area measured in centimeters at rest, on Valsalva maneuver, and on pelvic floor muscle contraction. All measures transformed into Z-score	POP-Q stages I, II, III dichotomized as present or absent	AUCs of 0.65 and 0.69 for hiatal area at Valsalva and hiatal length at Valsalva respectively for POP stage I. AUC of 0.77 and 0.72 for hiatal area at Valsalva and hiatal length at Valsalva respectively for POP stage II. AUC of 0.94 for hiatal area at Valsalva and 0.86 for hiatal length at Valsalva for POP stage III. ROC illustrated that no ideal cut-off to distinguish between “normal” and POP stage I. If we consider women who had no POP or had asymptomatic POP (stage I) to be “normal” and those with symptomatic POP (stage II or more) to be “abnormal,” the ROC proposes a maximal hiatal area of 20 cm^2^ or an AP of 6 cm (Z-score of 1.0) as cut-off with sensitivity of 79% and specificity of 65% for hiatal area and sensitivity of 71% and specificity of 58% for hiatal length
Wen and Zhou [51]	Measures of levator plate	AP diameter was measured in the midsagittal plane as the minimal distance between the hyperechoic inferoposterior aspect of the pubic symphysis and the hyperechoic anterior border of the pubovisceral muscle. The hiatal area was measured in the minimal axial plane with minimal dimensions	POP-Q stage II or higher defined as clinically significant prolapse. Symptomatic POP is defined as subjective symptoms of a bulge	AUCs 0.63 for the AP diameter and 0.66 for the hiatal area according to POP-Q stage II and higher. AUCs 0.75 for the AP and 0.82 for the hiatal area against prolapse symptoms. A cut-off of 6.0 cm for the AP diameter against POP-Q stage II and higher yielded sensitivity of 73% and specificity of 52%. A cut-off of 20 cm^2^ for the hiatal area against POP-Q stage II and higher produced sensitivity of 76% and specificity of 54%. For prolapse symptoms, the cut-off of 6.0 cm for the AP diameter had sensitivity of 74% and specificity of 64%, and the cut-off of 20 cm^2^ for the hiatal area had sensitivity of 78% and specificity of 68%
Wu et al. [49]	Topographic	Uterine descent was measured relative to the horizontal line positioned through the posteroinferior margin of the symphysis pubis on maximal Valsalva	POP-Q stages I and above and stage II and above for the apical compartment. Symptomatic POP is defined as subjective symptoms of the bulge	AUCs for POP symptoms 0.75. AUCs for POP-Q stage I and above 0.83; for POP-Q stage II and above 0.85. Cut-off values: 4.79 mm above the symphysis pubis for POP symptoms; cut-off 6.63 mm above the symphysis pubis for POP-Q stage I +; cut-off 8.42 mm below the symphysis pubis for POP-Q stage II+. Likelihood ratio + 1.91 for symptoms; 2.72 for POP-Q stage I+; 8.67 for POP-Q stage II+. Likelihood ratio 0.34 for symptoms; 0.25 for POP-Q stage I+; 0.39 for POP-Q stage II+
Braverman et al. [12]	Topographic and levator plate	Maximal caudal displacement of pelvic organs in relation to the symphysis pubis on maximal Valsalva maneuver supine and standing. Hiatal area on maximum Valsalva maneuver	Clinically significant prolapse defined as POP-Q stage II or higher in the anterior and posterior compartments or stage I or higher in the central compartment	AUCs for the anterior compartment: 0.64 supine and 0.67 standing; for uterine descent: 0.64 supine and 0.67 standing; for the posterior compartment: 0.57 supine and 0.6 standing; for the hiatal area: 0.68 supine and 0.72 standing
Shekand Dietz [89]	Topographic	Apical descent was measured relative to the posteroinferior margin of the symphysis pubis	Symptomatic POP is defined as subjective symptoms of the bulge	AUC 0.68 with sensitivity 0.7 and specificity 0.57 for a cut-off of −15 mm (15 mm above the symphysis pubis). AUC 0.74 with sensitivity 0.7 and specificity 0.64 for a cut-off of −15 mm (15 mm above the symphysis pubis) after patients with anterior- and posterior-dominant prolapse were excluded
Zhuang et al. [50]	Levator plate	Levator–urethra gap, levator–symphysis gap, and puborectalis attachment	MRI diagnosis of unilateral or bilateral avulsion	AUC 0.906 by levator–urethra gap for the diagnosis of levator avulsion with a cut-off of 23.65 mm (sensitivity, 92%; specificity, 95%). AUC 0.906 by levator–symphysis for the diagnosis of levator avulsion with a cut-off of 28.7 mm (sensitivity, 84.6%; specificity, 69.7%)

*AP* anteroposterior, *POP* pelvic organ prolapse, *POP-Q* Pelvic Organ Prolapse Quantification, *BW* Baden–Walker, *ROC* receiver-operating characteristic, *AUC* area under the curve

Receiver-operating characteristic curves appear to be similar for both anterior and posterior compartments, although the relationship between organ descent and symptoms was slightly stronger for the anterior compartment. Topographic markers appear to detect symptoms of POP only slightly better than the marker of the levator plate. ROC analysis indicates that the probability of transperineal ultrasound (TPUS) in detecting symptomatic prolapse increases with increasing POP stage [49]. Studies reporting the diagnostic value of TPUS in an Asian Chinese population report slightly better diagnostic accuracy than studies originating in Australia. The sensitivities and specificities of TPUS in detecting symptoms of POP and physical evidence of POP range from high 60 to low 80 and depend on the population, definition of POP, POP compartment assessed in the study, and POP severity [47, 49–53]. Only one study reported a likelihood ratio, the parameter that allows the assessment of an individual patient’s probability of having POP [49]. The likelihood ratio (LR) describes the chance of a positive sonographic marker being expected in a patient with POP compared with the likelihood of the same result being expected in a patient without POP. LR close to 1 means that the test result does not appreciably change the likelihood of POP. Ideally, the LRs should be either above 10 or below 0.1 to provide strong evidence to rule POP in or out. The positive LRs were 1.91 for ultrasound detecting symptoms of POP, 2.72 for detecting ≥ POP-Q stage I, and 8.6 for detecting ≥ POP-Q stage II. The negative LRs were 0.34, 0.25, and 0.39 respectively, indicating that ultrasound performs marginally better in ruling in advanced-stage POP than ruling in it out. LRs showed a rather small effect in predicting symptoms of POP or less advanced POP. This study focused on detecting uterine prolapse only [49].

##### Conclusion

The standardized technique of TPUS for POP detection is generally accepted; however, the accuracy of transperineal ultrasound in detecting symptoms or the anatomical finding of POP is moderate at best. The accuracy slightly improves in the standing position and with increasing POP severity. TPUS assessment in the standing position can be performed in cases where false-negative findings are suspected after a supine assessment. TPUS findings need to be interpreted with caution in patients with milder forms of POP. Reports on the diagnostic accuracy of TPUS findings are limited to two specific populations, as most studies originated in Australia and Asia. There is significant variation in diagnostic cut-offs for POP detection with regard to uterine descent between the two populations.

#### Other Types of Ultrasound

Six studies evaluated the diagnostic significance of alternative ultrasound modalities for the diagnosis of POP. Three studies attempted to assess endoanal ultrasound, two studies commented on endovaginal ultrasound, and one study explored the value of transabdominal ultrasound.

##### Endoanal Ultrasound

**Fig. 8:**
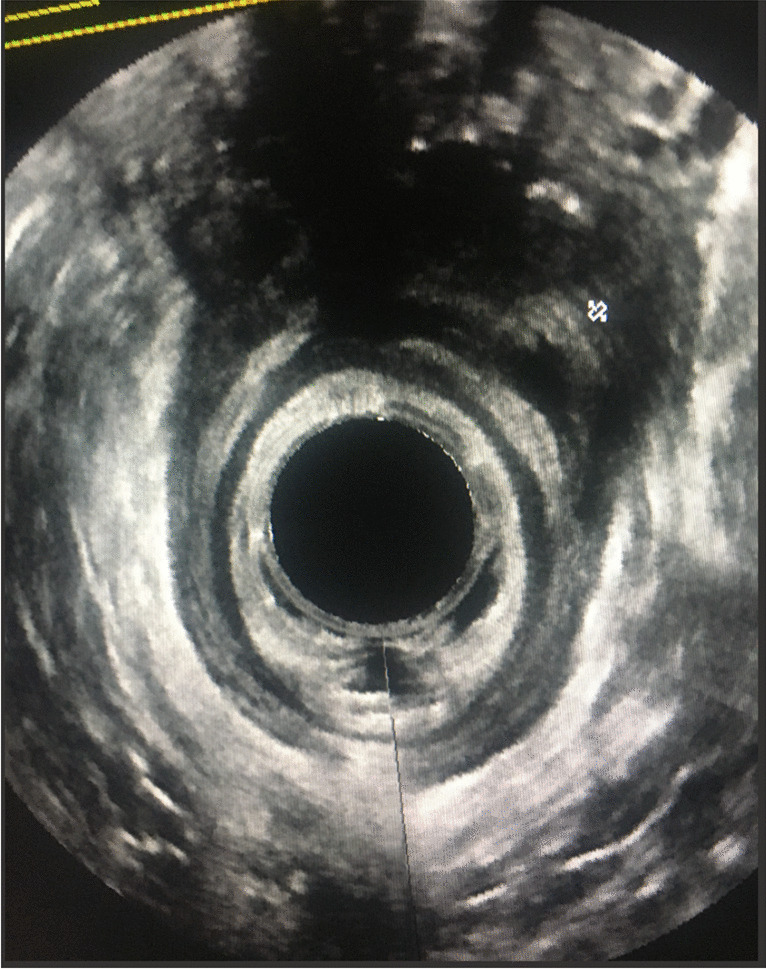
Endoanal ultrasound with internal and external anal sphincter defect

All three studies utilizing endoanal ultrasound in the diagnosis of POP were performed among patients awaiting surgical intervention for POP (Fig. 8). The technique was not standardized, and the ultrasound evaluations were performed in lithotomy, lateral decubital, or supine positions with the use of a tilting table. One study used a Foley balloon inserted in the bladder to better delineate the anterior compartment POP [54]. As POP-Q is not commonly accepted among colorectal surgeons as a gold standard reference for POP diagnosis, the diagnostic accuracy of endoanal ultrasound was described in correlation with intraoperative findings during POP surgery. Vierhout et al. focused on the posterior compartment and identifying enteroceles [54]. The authors described a sonographic marker of peristaltic loops of small bowel protruding into the vagina as evidence of an enterocele herniation of the pouch of Douglas. They concluded that rectal ultrasonographic findings were in good accordance with intraoperative anatomical diagnosis of enterocele. In 27 out of 29 patients (93%), when an enterocele was diagnosed by endoanal ultrasound, it was confirmed during surgery. Karaus et al. also focused on diagnosing enterocele, and defined an enterocele sonographic marker as the opening of a cul-de-sac into the vagina [55]. Only 4 patients out of the entire cohort of 17 underwent surgery, and enterocele was confirmed for all of them. Minagawa et al. explored all compartments for prolapse using the endoanal technique and compared ultrasound against intraoperative findings in 31 patients. This study used postural change from supine to standing position on a tilting table and defined any descent of the bladder neck from the baseline supine position as cystocele, descent of the vaginal vault of more than 3 cm as apical prolapse, and descent of the posterior vaginal wall of more than 1 cm as rectocele. The authors reported sensitivity, specificity, and accuracy of 90%, 83%, and 74% for anterior, apical, and posterior prolapse respectively [56].

##### Endovaginal Ultrasound

Two studies describing endovaginal ultrasound use in POP diagnosis focused on two different aspects of POP. Lone et al. aimed to assess if ultrasound findings can aid in diagnosing additional POP, which would lead to a change in planned surgical intervention. They concluded that clinical examination is better at diagnosing cysto-urethrocele, rectocele, and uterine prolapse. In addition, endovaginal ultrasound also diagnosed two intussusceptions and a combined enterocoele and intussusception in one woman, but overall did not have an impact in the planned management approach [4]. Athanasiou et al. reported on the ability of endovaginal ultrasound to assess the levator hiatal area and correlated it with POP-Q findings. The authors concluded that the hiatal area, and not levator thickness, is in strong correlation with POP-Q measurements in all compartments [57].

##### Transabdominal Ultrasound

One study that utilized the transabdominal ultrasound technique, attempted to use it to diagnose paravaginal defects. The role of the paravaginal defect in POP is debated in the literature and the defect itself is not adequately reflected by the POP-Q measuring system [58]. The authors attempted to evaluate if the lateral bladder base “sagging” below level of the central bladder correlates with the physical examination diagnosis of a paravaginal defect established with the use of ring forceps reducing the apical prolapse. The authors concluded that the sonographic paravaginal defects identified in this study were artificially created by the ultrasound technique, utilizing a balloon placed in the vagina to enhance vaginal forces. Hence, this technique cannot accurately diagnose paravaginal defects.

##### Conclusion

The diagnostic value of endovaginal and endoanal ultrasound is limited to the detection of enterocele, likely because insertion of the probe into the vaginal or rectal cavity distorts the descent of the prolapsing organs by adding a space-occupying effect. There is no adequately described transabdominal ultrasound technique that can aid the diagnosis of prolapse.

### Head-to-Head Comparison of Different Imaging Techniques

Seven studies included a second imaging technique in the assessment of patients’ POP, while attempting to define the value of the index imaging technique in POP diagnosis. Some of the studies used another technique to define cases of POP, as they found POP-Q alone inadequate as a standard reference for testing accuracy. For example, Van Gruting et al. used a composite reference standard to define POP + cases: DCP, MRI, and physical examination findings needed to agree in order to meet the definition of the positive reference case for POP [19]. Other studies provided insight into how the techniques compare in POP detection. Martellucci and Naldini reported good correlation between DCP and ultrasound when assessing patients with rectocele (88% agreement) [20]. Beer-Gabel et al. demonstrated that DCP and the ultrasound techniques showed good concordance for the diagnosis of enterocele [59].

Broekhuis et al. aimed to evaluate agreement between MRI and TPUS in detecting prolapse in all compartments and concluded that the two imaging techniques correlate moderately to well only in the anterior compartment [17]. Barakat et al. performed a true blinded head-to-head comparison of MRI and TPUS in detecting POP defined via POP-Q, and concluded that both techniques perform similarly in POP detection in all three compartments, but these findings are limited only to high-grade (POP-Q stage III and IV) POP [6]. Finally, Zhuang et al. focused on the ability of MRI and TPUS to assess levator avulsion and reported 92% agreement, with a kappa of 0.79 between the two techniques [50].

#### Conclusion

Transperineal ultrasound has a moderate correlation with DCP in diagnosing enteroceles. MRI and TPUS can be used interchangeably in the diagnosis of levator avulsion but differ in their detection of POP in individual compartments, especially in cases of mild POP.

### Role of Imaging Techniques in the Clinical Management of POP

In 6 studies (3 on MRI and 3 on TPUS) the authors attempted to evaluate the role of imaging in surgical or conservative POP management.

#### Magnetic resonance imaging

Van der Weiden et al. reported on MRI measurements before and 6 months after sacrocolpopexy and concluded that MRI only revealed significant improvement for the apical compartment, with no correlation between changes in MRI measurements, POP-Q measurements, and validated questionnaires [35]. Siegmann et al. reported on the MRI assessment of patients before and after pelvic floor repair with transvaginal mesh, demonstrating clinically occult POP cases in 73.3% of patients at 3 months after repair, but this finding did not correlate with clinical symptoms [37]. Attenberger et al. focused specifically on the ability of MRI to provide additional information not evident according to the physical examination [27]. The latter study evaluated if the MRI diagnosis of POP had an impact on the treatment strategy or altered the surgical procedure: the treatment plan was changed in 13 out of 50 cases (26%). In 12 cases, an enterocele was diagnosed by MRI, but was not detected on physical examination. In 4 cases, an enterocele and in 2 cases a rectocele were suspected clinically but were not confirmed by MRI. The study did not have a comparison group and did not provide any data on patient-centered surgical outcomes.

#### Transperineal Ultrasound

Lone et al. studied whether baseline assessment with ultrasound in addition to routine physical examination added diagnostic value leading to management change in patients with POP in a prospective cohort study with normal controls [4]. Although TPUS enhanced the visualization of additional pelvic floor abnormalities and identified a higher number of additional ultrasound pathological conditions in the POP group (11.3% enteroceles and 3.4% intussusceptions), it did not lead to a change in the clinical treatment plan, as the majority of abnormalities (mainly enteroceles) were small. Huang et al. assessed the role of TPUS in patients undergoing transvaginal mesh and native tissue repair surgery for anterior compartment POP and commented on the ability of ultrasound parameters to predict surgical failure [60]. They reported that preoperatively, patients with and without POP recurrence were similar in the POP-Q staging and ultrasound measures of levator hiatus. On 12-month postoperative ultrasound, patients with POP recurrence in the anterior compartment demonstrated a higher rate of complete levator avulsion (OR 14.2; CI 4.8–42.2). Gillor et al. performed a similar study focusing on sonographic outcomes of posterior compartment correction with and without mesh augmentation [46]. They reported that clinical recurrent posterior vaginal wall prolapse (defined as point Bp ≥  −1) was seen in 20% of patients, whereas POP diagnosed by ultrasound (defined as descent of the rectal ampulla ≥ 15 mm below the symphysis pubis) was noted in 12% of patients. An additional 6% exhibited enterocele findings on ultrasound (diagnosed if an enterocele sac was seen at or below the level of the symphysis pubis on imaging) [46]. There was no difference in sonographic levator avulsion between the mesh-augmented and native tissue repair groups in this study [46]. In those without significant posterior compartment descent on clinical examination, a substantial minority still showed a “true rectocele,” (defined as defect of the rectovaginal septum). This was the case after both mesh (29%) and native tissue repair (18.5%).

## Conclusion

In comparison trials, no imaging modality appeared superior to another. Overall, the value of diagnostic imaging in POP management remains unclear and understudied.

## Recommendations

- Computerized tomography and fluoroscopic imaging such as defecography should not be used routinely to diagnose POP, as there are not enough well-designed studies reporting on their accuracy for POP detection.
- The value of defecography is in the detection of enterocele and intussusception.
- The value of MRI in diagnosing multicompartment POP is unclear, as studies reporting diagnostic accuracy are very heterogeneous in technique, reference lines used, and definitions of POP reference standards.
- Exposed vaginal length with a cut-off value of 2.9 cm can be used to detect large rectocele on MRI with good accuracy.
- Transperineal ultrasound can be used to detect clinically significant POP and symptomatic POP with moderate accuracy. Accuracy decreases as POP-Q stages decrease.
- Cut-off values for uterine POP are population specific and need to be used with caution in populations other than white Australian or Asian Chinese women.
- The value of endovaginal and endoanal ultrasound in prolapse detection and quantification is limited to the detection of enteroceles.
- Both MRI and TPUS can be used in the diagnosis of levator ani avulsion.
- High-quality studies should be performed to evaluate the utility of imaging in the clinical management of POP.

## Discussion Points

The intended use of imaging test can be diagnosis, screening, staging, monitoring, surveillance, prediction, or prognosis. This narrative review focuses on the diagnostic value of different imaging modalities in POP. Our working group identified a significant gap in the current literature surrounding imaging use. There is a lack of well-designed studies with clear definitions of reference standard defining POP as disease. Largely, it is stemming from the lack of consensus of what defines “clinically significant POP.” Imaging techniques appear to perform moderately well in the diagnosis of advanced POP stages, but those POP cases are easily diagnosed on pelvic examination. An ideal study would be a large cohort focusing on low-grade POP, where the “clinically significant prolapse” is diagnosed as a combination of pelvic examination evidence of POP and the presence of symptoms determined by validated questionnaires. The studies with a similar design will need to be repeated in diverse patient populations. Only after this evidence is obtained can we gain a better insight into the diagnostic abilities of different imaging modalities.

## Abbreviations

AUGS: American Urogynecologic Society
ICS: International Continence Society
IUC: International Urogynecological Consultation
IUGA: International Urogynecological Association
POP: Pelvic organ prolapse
POP-Q: Pelvic Organ Prolapse Quantification
SGS: Society for Gynecologic Surgeons
